# Sense of Place and Health in Hamilton, Ontario: A Case Study

**DOI:** 10.1007/s11205-012-0065-1

**Published:** 2012-05-04

**Authors:** Allison Williams, Peter Kitchen

**Affiliations:** 1 School of Geography and Earth Sciences, McMaster University, 1280 Main Street West, Hamilton, ON L8S 4K1 Canada; 2 McMaster Institute of Environment & Health, McMaster University, Hamilton, ON Canada

**Keywords:** Sense of place, Health, Neighbourhood, Socio-economic status

## Abstract

The concept of sense of place has received considerable attention by social scientists in recent years. Research has indicated that a person’s sense of place is influenced by a number of factors including the built environment, socio-economic status (SES), well-being and health. Relatively few studies have examined sense of place at the neighbourhood level, particularly among communities exhibiting different levels of SES. This article investigates sense of place among three neighbourhood groups in Hamilton, Ontario representing areas of low, mixed and high SES. It analyses data from a 16-point sense of place scale derived from the Hamilton Household Quality of Life Survey carried out in 2010–2011 among 1,002 respondents. The paper found that sense of place was highest among residents of the high SES neighbourhood group as well as among home owners, people residing in single-detached homes, retired residents and those living in their neighbourhood for more than 10 years. From a health perspective, the paper found that a strong association existed between sense of place and self-perceived mental health across the three neighbourhood groups. Furthermore, by way of regression modeling, the paper examined the factors influencing health-related sense of place. Among the sample of respondents, a strong connection was found between housing, particularly home ownership, and high levels of health-related sense of place.

## Introduction

People simultaneously experience numerous risk and protective factors reflecting the sum total of natural, built and socio-cultural environmental factors. Research has indicated that socio-economic deprivation is associated with health and that significant differences exist within urban areas (Ross et al. 2004; Heisz and McLeod 2004; CPHI 2006). With about 80 % of Canada’s population living in urban areas, it is important to investigate these issues, especially at the neighbourhood level. We recognize that physical and social environments impact health, but know very little about how different aspects of these local environments interact in influencing health (Macintyre et al. 2002). One way to understand these complex processes is to examine the mechanisms or pathways through which place and the social relations within it shape the health status of individuals and populations. One novel conceptual approach that is missing from current studies examining health effects of local environments is the subjective meaning and importance that individuals give to where they reside. In other words, the perceptions residents have of their own environments, encompassing social and structural features; this is known as the place-based construct named *sense of place*.

We have adopted the following conceptualization of sense of place in this research, as articulated in the edited volume titled *Key Thinkers on Space and Place* (Hubbard et al. 2004, p. 351) which defines sense of place as a geographical concept “intended to describe the particular ways in which human beings invest their *surroundings with meaning*”. The Dictionary of Human Geography (2009) recognizes that sense of place refers to “the attitudes and feelings that individuals and groups hold *vis*-*à*-*vis* the geographical areas in which they live. It further commonly suggests intimate, personal and emotional relationships between self and place” (Wylie, p. 676). Sense of place is, therefore, simultaneously understood in this research as pertaining to geographical place, social community/environment and having psychoanalytic meaning. Recognizing that individual perceptions of *sense of place* can apply to a wide range of settings (e.g. cottages or homeland), our interest is in the neighbourhoods where people ‘live’ and not necessarily where they ‘work’ or ‘play.’

The aim of this research is to assess the socio-demographic characteristics of sense of place in three divergent neighbourhoods in Hamilton, Ontario, Canada. In so doing, this paper answers the following research question: *How does sense of place vary between residents of three contrasting neighbourhoods and how does sense of place relate to health outcomes in these neighbourhoods*? The impetus for this research has been the dearth of empirical work on sense of place particularly at the neighbourhood level. This paper will elaborate on the results of these questions while proposing policy and program implications that can be implemented at the neighbourhood level.

Hamilton is a mid-sized Canadian city located in the southern portion of the province of Ontario, about 75 km west of Toronto. In 2011, it had a population of 520,000. Throughout its history, Hamilton has served as an important industrial centre active in steel production, manufacturing and transportation. In recent years, economic restructuring has resulted in the loss of thousands of industrial jobs and growth in the service and knowledge based sectors, particularly health and education. However, economic change has resulted in Hamilton’s once robust core experiencing decline. A socio-economic divide is evident among residents with several neighbourhoods in the city’s central and eastern sections suffering from high levels of poverty and disadvantage. In these areas, the standard of living has lowered, child poverty has increased and more families are using food banks.

## Place-Based Research and the Population Health Perspective

Systematic investigations of relationships between compositional (individually-based) and contextual (place-based) characteristics and health status have yet to determine fully the independent importance of contextual factors, as well as how they interrelate with one another in informing health status. The centrality of place to already known pathways between social circumstances and health (e.g. income inequalities and health, and social support and health) further reinforces the need for greater research on the role of place in the social production of health. For example, in our own work on urban quality of life, residents of poor neighbourhoods were found to have compositional characteristics (e.g. income level, employment status) associated with more positive self-reported health, while for residents in wealthier neighbor-hoods, contextual characteristics (e.g. feeling safe/secure) figured more prominently, suggesting a need for nuanced strategies rather than a ‘one size fits all’ approach (Muhajarine et al. 2008).

While multi-level studies of persons and place are increasing, most ignore a potentially important factor: an individual’s perceptions of and relationship to his or her place of residence, or their sense of place. Sense of place may be seen as a key construct in place-based health research, as it provides a conceptual link between the exogenous area-based variables and the internal biological processes and systems in individuals. Area-level variables, however conceptualized, do not directly influence biological systems; individuals’ perceptions of and relationship to their local environment (whether relative to people living in the community or in relation to physical amenities, resources or services available) represent key mechanisms through which attributes of the local area begin to manifest in individual biological systems. While the importance of individual response to the environment (both social and physical) is not new and has been an enduring explanatory mechanism in several areas of research investigation (e.g. income inequality, social capital and social cohesion), the specific application and integration of *sense of place* to place-specific health research has not been commonly done.

Geographers have incorporated the construct of sense of place into health research. The work conducted on health and the meaning of place (Gesler 1992, 1993; Williams 1998, 1999; Kearns 1991, 1995) has directed attention to sense of place and its role in health. We also have some evidence that sense of place contributes to community-level pathways and processes that positively influence health (Warin et al. 2000; Theodori 2001). The perceptions and meaning ascribed to place can also have negative connotations. For example, ‘neighbourhoodism’ is a term used to describe the stereotyping in media and public attitudes of poor, ghettoized communities and suburbs. The negative attributes of these areas are often attributed to people living there who, in turn, often internalize these features as being partly a reflection of their own lack of self-worth, leading to poorer health or quality of life (Williams et al. 2002). A positive image of neighbourhood, in turn, may have a salutary effect by enhancing personal attitudes, behaviours and self-concept, and thereby health and quality of life (Kearns et al. 2000; Meegan and Mitchell 2001; Healey 1998).

Health geographers have examined related concepts such as the relationship between perceptions of specific environments and health. In James and Eyles’ (1999) exploratory study of perceptions of health and the environment among men and women in lower and higher status areas, they found that both genders referred to health and environment as connected concepts. Men, however, referred to this connection more than women and lower income participants noted environment-related health problems more often but in less detail. Wakefield et al.’s (2001) study of health risk perception and community action revealed that those interviewed tended to report adverse effects on their health if air pollution was visible. Wakefield et al. (2001) also found that strong levels of place attachment were found to be a necessary (but not sufficient) condition for community action and that social capital may be beneficial in overcoming feelings of powerlessness to address environmental issues, ultimately improving one’s health. Similarly, Luginaah et al. (2002) reported that odor perceptions and annoyance with a petroleum factory were key mediators in reporting illness. In a study of four socially contrasting neighbourhoods in Glasgow, Ellaway and Macintyre (2001) found that, after accounting for individual differences, neighbourhood of residence was associated with perceptions of problems and neighbourhood cohesion in the area and these characteristics, in turn, were associated with self-assessed health, mental health and recent symptoms.

A number of studies in other disciplines have examined the importance of how one perceives certain aspects of the local environment and have found significant effects on health. For example, Collins et al. (1998) found that, among African-American mothers, perceptions of their residential environment (including police protection, personal safety, cleanliness and quietness) were associated with very low birth weight outcomes even after controlling for maternal behaviours such as alcohol use and cigarette smoking. Ewart and Suchday’s (2002) application of the City Stress Inventory (CSI) found that CSI subscales were associated with elevated chronic levels of depression, anger, attitudes of interpersonal distrust and low self-esteem. Similarly, in Fuller et al.’s (1993) examination of objective and subjective housing conditions and well-being, they concluded that objective housing conditions were not associated with many of their 10 measures of health but that housing satisfaction was significantly related to half of their measures of health.

Researchers have considered a number of related constructs, such as place attachment (e.g. Altman and Low 1992; Hidalgo and Hernandez 2001), community satisfaction (e.g. Bardo and Bardo 1983) and sense of community (e.g., Glynn 1981; Chavis et al. 1986; Nasar and Julian 1995; Robinson and Wilkinson 1995; Pendola and Gen 2008). Sense of place emanates from the experiences and perceptions of individual residents. By contrast, other place-related concepts, such as place identity, are often shaped by external forces such as the views or stereotypes of those living outside of the neighbourhood.

We recognize that *sense of place* is very likely a multi-dimensional (rather than uni-dimensional or bi-dimensional) and dynamic construct in that the nature of the dimensions could vary over time and place as a function of the characteristics of an (a) individual, and (b) neighbourhood and larger local community. For example, the nature of a person’s individual *sense of place* may be a complex combination of awareness of neighbourhood characteristics and the individual’s subjective reaction to those characteristics that he or she believes are salient community features. The subjective reaction could be positive, neutral or negative. Similarly, different neighbourhood settings could necessitate varying dimensions and the relative importance of those dimensions could also change based on the salient features of the nieghbourhood, the surroundings of the neighbourhood, and the amenities within the neighbourhood.

As noted, sense of place has not only been examined in a wide variety of disciplines, but also in a number of spatial contexts and scales including: communities (Taylor and Townsend 1976; Eyles 1985; Hummon 1992; Howley et al. 1996; Butz and Eyles 1997; Hay 1998; Derr 2002; Pretty et al. 2003); ethnic enclaves (Mazumdar et al. 2000); public places (Oritz et al. 2004); and regions (Shamai 1991; Shamai and Ilatov 2004). Despite the plethora of studies, there is a dearth of empirical research specific to sense of place at the neighbourhood level, particularly in Canada.

## Data and Methods

This paper employed data from the Hamilton Household Quality of Life survey carried out by McMaster University between November 2010 and March 2011. A total of 1,002 households responded to the survey, which posed a series of questions relating to neighbourhood quality of life and health. The survey included 16 questions that measure a person’s sense of place (see Table 1). The survey targeted three neighbourhood clusters in Hamilton representing areas of different socio-economic status (SES)—low SES (Lower City), Mixed SES (Central) and high SES (Southwest Mountain). The criteria for selecting the neighbourhood clusters were as follows: (1) each neighbourhood had a population greater than 1,000, (2) neighbourhoods in each cluster were contiguous and represent identifiable boundaries and (3) each cluster represented socio-economic conditions and important geographic locations within Hamilton as determined by the 2006 census. The objective was to collect data on approximately 300–350 households in each cluster. A random sample of telephone numbers (associated with unique households) was used as the basis for the sampling frame; 3,599 households were contacted with a response rate of 28 % (*n* = 1,002).

**Table 1:** Hamilton Household Quality of Life Survey Sense of Place Module: ‘Short Scale’ (16 items)

Survey questions and coded responses^a^
*Neighbourhood rootedness*
1. There’s no other neighbourhood you would rather live in (1. strongly agree, 2. somewhat agree, 3. neutral, 4. somewhat disagree, 5. strongly disagree)
2. How rooted do you feel in your neighbourhood? (1. very rooted, 2. fairly rooted, 3. neutral, 4. not very rooted, 5. not at all rooted)
3. You would like to stay in your neighbourhood as long as your health allows you to do so (1. strongly agree, 2. somewhat agree, 3. neutral, 4. somewhat disagree, 5. strongly disagree)
4. If you were to live somewhere else, it would be difficult to move away from your neighbourhood (1. very true, 2. partly true, 3. neutral, 4. not very true, 5. not at all true)
*Neighbourhood sentiment*
5. Your neighbourhood means a great deal to you (1. strongly agree, 2. somewhat agree, 3. neutral, 4. somewhat disagree, 5. strongly disagree)
6. You feel at home in your neighbourhood (1. strongly agree, 2. somewhat agree, 3. neutral, 4 somewhat disagree, 5. strongly disagree)
7. How connected do you feel to your neighbourhood? (1. very connected, 2. fairly connected, 3. neutral, 4. not very connected, 5. not at all connected)
8. How much do you like your neighbourhood? (1. a great deal, 2. a fair amount, 3. neutral, 4. not very much, 5. not at all)
*Neighbours*
9. You know many of your neighbours on a first name basis (1. very true, 2. partly true, 3. neutral, 4. not very true, 5. not at all true)
10. How often do you participate in social activities with your neighbours? (1. all the time, 2. often, 3. sometimes, 4. hardly ever, 5. never)
11. There are people in your neighbourhood who you think of as close friends (1. strongly agree, 2. somewhat agree, 3. neutral, 4. somewhat disagree, 5. strongly disagree)
12. If you had to leave, how many of your neighbours would you miss? (1. many of them, 2. some of them, 3. neutral, 4. hardly any of them, 5. none of them)
*Environment/health*
13. Green space availability in your neighbourhood positively influences your health (1. strongly agree, 2. somewhat agree, 3. neutral, 4. somewhat disagree, 5. strongly disagree)
14. Environmental problems in your neighbourhood influence your health (5. strongly agree, 4. somewhat agree, 3. neutral, 2. somewhat disagree, 1. strongly disagree)
15. Social problems in your neighbourhood (e.g. racism, violence) influence your health (5. strongly agree, 4. somewhat agree, 3. neutral, 2. somewhat disagree, 1. strongly disagree)
16. The personal safety of yourself and your family in your neighbourhood affects your health (5. strongly agree, 4. somewhat agree, 3. neutral, 2. somewhat disagree, 1. strongly disagree)

^a^The answers to questions 14, 15 and 16 are reverse coded to maintain consistency in the magnitude of responses (positive or negative) across the 16 items in the short scale

As discussed in detail in our earlier work (Williams et al. 2010), few studies have attempted a quantitative analysis of sense of place at the neighbourhood level. In our empirical study of sense of place, we used a fully tested and validated survey instrument to collect data at the neighbouhood level (Williams 2008). The data analysis, as explained in detail in Williams et al (2010) contained the development and application of a *neighbourhood sense of place score* based on the survey data. First, an inductive search for common patterns in the survey data set was conducted, using principal components analysis (PCA) on the 46 variables to identify broad dimensions of neighbourhood sense of place. Next, descriptive statistics (mean, standard deviation, and *Z* scores) were used to measure the level of sense of place among respondents according to selected characteristics (e.g. gender, age, marital status). Inferential statistics (*t* tests) were also used to examine differences between groups on the selected characteristics. The PCA and the descriptive statistics were employed to create two neighbourhood sense of place scores: a ‘long scale’ based on 46 survey items, and a ‘short scale’ based on 16 items. This short scale of 16 items (see Table 1) was used again in the current study of Hamilton given that the ‘short scale’ was deemed the most appropriate for future research on sense of place as it is relatively simple to calculate and can be readily applied to the neighbourhood scale (Williams et al 2010). The 16 items were grouped equally into four sense of place factors corresponding to the results of the PCA: ‘Rootedness’, ‘Sentiment’, ‘Neighbours’, ‘Environment/Health’. As demonstrated in Table 1, each factor contains four questions where the responses are on a 5-point scale with 1 representing a positive response to the question (e.g. ‘strongly agree’; ‘very true’) and 5 representing a negative response (e.g. ‘strongly disagree’; ‘not true at all’).

To determine the sense of place score, we summed the values of the raw data of each respondent (*n* = 1,002) and then summed the total across the four factors as a percentage. The equation is presented below:

$$ {\text{Nghd}}\,{\text{SoP}} = ( 20 - \sum {\text{Factor1}}) + ( 20 - \sum {\text{Factor2}}) + ( 20 - \sum {\text{Factor3}}) + ( 20 - \sum {\text{Factor4}})/ 6 4\times 100 $$

The sum of each individual factor ranges from a low of 4 (positive sense of place) to a high of 20 (weak sense of place). A low score of 4 (denoting positive sense of place) is achieved because the answers given by the respondent on each of the four questions were coded 1 (e.g. ‘strongly agree’ or ‘very true’), the highest possible rating out of 5. Conversely, a high score of 20 (denoting weak sense of place) is achieved because the answers given by the respondent on each of the four questions were coded 5 (e.g. ‘strongly disagree’ or ‘not true at all’), the lowest possible rating out of 5. The problem with this approach is that a low cumulative score means a positive outcome and a high cumulative score means a negative outcome, possibly leading to confusion by users of the short scale. As a result, for better interpretation, the scores on each factor were reversed so a high number represents positive sense of place and a low number represents weak sense of place. This was achieved by simply subtracting the sum of the 4 factors from 20 to create a scale from 0 to 16 with 0 representing the worst possible sense of place and 20 representing the best possible sense of place.^1^ The total sum of the 4 factors was then divided by 64 (the maximum score) to create a score represented as a percentage with values close to 100 indicating a strong sense of place. For example, using the equation above, the neighbourhood sense of place score for survey respondent 001 was calculated as follows:

$$ \begin{aligned} {\text{Nghd}}\,{\text{SoP}} & = ( 20 - 4) + ( 20 - 4) + ( 20 - 5) + ( 20 - 8)/ 6 4\times 100 \\ {\text{Nghd}}\,{\text{SoP}} & = ( 1 6+ 1 6+ 1 5+ 1 2)/ 6 4\times 100 = 9 2. 2\\ \end{aligned} $$

Table 2 shows the mean sense of place scores for the entire survey sample across the three neighbourhood groups (*n* = 1,002). In order to look more closely at these scores in the comparatively vulnerable Lower City, the low-SES neighbourhood cluster (*n* = 293) was examined in greater detail (Table 3). In order to determine whether significant differences exist among the categories making up each of the variables, a one-way analysis of variance (ANOVA) was conducted. ANOVA is a procedure used to make tests comparing the means of several populations. By using a one-way ANOVA test, we are analyzing only one variable, for example, the effect of neighbourhood residence on the sense of place score. The results of the one-way ANOVA for each variable, for both the citywide survey sample (Table 2) and, specifically, the Lower City (Table 3), determined some interesting results, which confirm much of our earlier work.

**Table 2:** Sense of Place in Hamilton: Short Scale based on 16 Items (all respondents *n* = 1,002)

Variable	Count	Mean sense of place score^a^	Mean difference ANOVA
*Neighbourhood cluster*			
1. Southwest Mountain	346	66.5	1-2** 1-3**
2. Central	363	63.0	2-1** 2-3**
3. Lower City	293	55.3	3-1** 3-2**
*Gender*			
1. Male	400	61.0	–
2. Female	602	62.6	–
*Age*			
1. 18–29	119	58.9	1-4**
2. 30–44	273	58.9	2-4**
3. 45–64	393	61.2	3-4**
4. 65 or over	217	69.0	4-1** 4-2** 4-3**
*Self-perceived health*			
1. Excellent/very good	549	63.9	1-2** 1-3**
2. Good	287	60.7	2-1**
3. Fair/poor	166	57.9	3-1**
*Self-perceived mental health*			
1. Excellent/very good	646	64.9	1-2** 1-3**
2. Good	261	59.0	2-1** 2-3**
3. Fair/poor	95	50.3	3-1** 3-2**
*Born in Canada*			
1. Yes	749	62.2	–
2. No	253	61.3	–
*Marital status*			
1. Married/common law	517	62.0	
2. Widowed/sep./divorced	257	64.2	2-3**
3. Single, never married	228	59.3	3-2**
*Education level*			
1. Less than high school	129	61.4	–
2. Completed high school	219	61.9	–
3. Some college or university	123	62.2	–
4. Completed college	214	62.1	–
5. Completed bachelor	194	61.9	–
6. Post-graduate/professional	123	62.4	–
*Employment status*			
1. Working full-time	468	61.3	1-3** 1-4**
2. Working part-time	103	58.0	2-3**
3. Retired	221	69.8	3-1** 3-2** 3-4**
4. Other (unemployed/student disability/homemaker)	210	57.3	4-1** 4-3**
*Lived in neighbourhood*			
1. Less than 5 years	279	54.9	1-2** 1-3** 1-4**
2. 5–10 years	259	60.7	2-1** 2-3** 2-4**
3. 11–19 years	257	66.3	3-1** 3-2**
4. 20 years or more	207	67.7	4-1** 4-2**
*Housing tenure*			
1. Own	692	63.6	1-2**
2. Rent	310	58.3	2-1**
*Dwelling type*			
1. Single detached	628	63.3	1-3** 1-4**
2. Semi-detached/duplex/row/townhouse	157	65.0	2-3** 2-4**
3. Low-rise apt/condo bldg	96	57.7	3-1** 3-2**
4. High-rise apt/condo bldg	121	54.5	4-1** 4-2**

^a^Mean Sense of Place Score for Entire Sample (*n* = 1,002) is 62.0

** Significant at 95 %

**Table 3:** Sense of Place in Hamilton: Short Scale based on 16 Items (Lower City respondents *n* = 293)

Variable	Count	Mean sense of place score^a^	Mean difference ANOVA
*Census tract*			
1. 51.00	80	54.1	–
2. 52.00	76	58.1	–
3. 60.00	46	55.5	–
4. 61.00	42	53.1	–
5. 67.00	39	56.3	–
*Gender*			
1. Male	114	56.8	–
2. Female	179	54.4	–
*Age*			
1. 18–29	30	53.0	1-4**
2. 30–44	76	53.6	2-4**
3. 45–64	136	53.7	3-4**
4. 65 or over	51	63.8	4-1** 4-2** 4-3**
*Self-perceived health*			
1. Excellent/very good	120	55.7	–
2. Good	97	56.1	–
3. Fair/poor	76	53.8	–
*Self-perceived mental health*			
1. Excellent/very good	167	58.3	1-3**
2. Good	90	53.8	2-3**
3. Fair/poor	36	45.3	3-1** 3-2**
*Born in Canada*			
1. Yes	231	55.8	–
2. No	62	53.4	–
*Marital status*			
1. Married/common law	128	54.8	–
2. Widowed/sep./divorced	81	57.4	–
3. Single, never married	84	54.1	
*Education level*			
1. Less than high school	65		–
2. Completed high school	70	55.6	–
3. Some college or university	39	56.7	–
4. Completed college	64	53.6	–
5. Completed bachelor	41	56.2	–
6. Post-graduate/professional	14	47.5	–
*Employment status*			
1. Working full-time	131	53.8	1-3**
2. Working part-time	32	50.2	2-3**
3. Retired	51	64.1	3-1** 3-2** 3-4**
4. Other (unemployed/student disability/homemaker)	79	54.3	4-3**
*Lived in neighbourhood*			
1. Less than 5 years	79	49.8	1-3** 1-4**
2. 5–10 years	80	51.2	2-3** 2-4**
3. 11–19 years	69	57.2	3-1** 3-4**
4. 20 years or more	65	65.3	4-1** 4-2** 4-3**
*Housing tenure*			
1. Own	203	56.4	–
2. Rent	90	52.9	–
*Dwelling type*			
1. Single detached	196	56.7	1-3**
2. Semi-detached/duplex/row/townhouse	41	56.9	2-3**
3. Low-rise apt/condo bldg	28	45.0	3-1** 3-2** 3-4**
4. High-rise apt/condo bldg	28	55.1	4-3**

^a^Mean Sense of Place Score for Lower City (*n* = 293) is 55.3

** Significant at 95 %

Next, we used ordinary least squares (OLS) linear regression to explore how geography and social-economic status influence sense of place. Two models were implemented. Model 1 consists of all respondents in the three neighbourhood clusters (*n* = 1,002). Model 2 includes respondents in the Lower City neighbourhood only (*n* = 293). A bootstrapping method was used to estimate the standard errors.

Finally, a second series of regression models were performed using logistic regression rather than OLS. In order to determine what influences the relationship between sense of place and self-assessed mental health, a dummy variable was generated. We defined high ‘health-related sense of place by generating a dummy variable where “1” indicates a sense of place score above the mean and self-assessed health and mental health as being ‘excellent/very good’. (In other words, a respondent considered to have high ‘health related sense of place’ must have both a higher than average sense of place and also have positive health outcomes). Next, we used logistic regression to examine the extent of the effect of socio-economic status and geography, as associated with the dummy variable. Bootstrapping methods were used to estimate the standard errors of the odds ratios.

## Results

The aim of this research was to assess the socio-economic characteristics of sense of place in three divergent neighbourhoods in Hamilton, Ontario. The paper posed the following research question: *How does sense of place vary between residents of three contrasting neighbourhoods and how does sense of place relate to health outcomes in these neighbourhoods*? To answer this question, two analytic stages were conducted: the calculation of the mean sense of place score, followed by an analysis of variance (ANOVA) for each variable for both the citywide survey sample (Table 2) and the Lower City (Table 3). The mean sense of place score for the citywide sample (*n* = 1,002) was 62, compared to that of the Lower City, which was 55.3. Analysis of each of these samples will be discussed in turn.

As shown in Table 2, the analysis of the entire sample yielded a number of significant results (see last column, Table 2), including two principal findings. The first was that there is a significant difference (*p* = 95 %) in sense of place scores across the three neighbourhood groups, with Southwest Mountain (high SES) having a significantly higher score than the Central (mixed SES) and Lower City (low SES). Further, the Central neighbourhood group was found to have a significantly higher sense of place score than the Lower City. Secondly, there was a significant difference (*p* = 95 %) in sense of place scores across two health outcomes: self-perceived health and self-perceived mental health. People who assessed their self-perceived health as ‘Excellent/very good’ had a significantly higher score (63.9) than those who assessed their health as ‘Good’ (60.7) or ‘Fair/poor’ (57.9). Additionally, those who assessed their mental health as ‘Excellent/very good’ had a significantly higher sense of place score (64.9) than those who assessed their mental health as ‘Good’ (59.0) or ‘Fair/poor’ (50.3). Significant differences were also found across age, with those aged 65 or over having a higher sense of place score than younger age groups. With respect to marital status, those who were ‘Widowed/separated/divorced’ had a higher score than did those who were ‘Single/never married’. Significant differences were also found in employment status, with ‘Retired’ people having a higher sense of place score than did those who were ‘Working’, either full-time or part-time, as well as those in the ‘Other’ category (unemployed/student/disability/homemaker). Those ‘Working full-time’ also had a significantly higher sense of place score than those in the ‘Other’ category.

Three remaining related factors were found to have significant differences: neighbourhood longevity, housing tenure and dwelling type. Long-term residents living in their neighbourhoods for 20 years or more years had significantly higher sense of place scores than did those who lived in their neighbourhood for ‘Less than 5 years’ or ‘5–10 years’. With respect to housing tenure, those who own their homes were found to have significantly higher sense of place scores than those who rented. With respect to dwelling type, those who lived in either ‘Single-detached’ or ‘Semi-detached/duplex/row/townhouse’ were found to have significantly higher scores than those who lived in either ‘Low’ or ‘High-rise’ apartment/condominium buildings.

The analysis of the sample comprising the Lower City (*n* = 293) yielded a number of significant results (see last column, Table 3) some of which were also apparent in the citywide sample. Similar to the citywide sample, there was a significant difference (*p* = 95 %) in sense of place scores across self-perceived mental health. Those who assessed their mental health as ‘Excellent/very good’ had a higher sense of place score (58.3) than those who assessed their mental health as ‘Good’ (53.8) or ‘Fair/poor’ (45.3). As in the larger city sample, significant differences were also found across age, with those aged 65 or over having a higher sense of place score (63.8) than younger age groups. As with the entire city sample, significant differences were also found in employment status, with ‘Retired’ having a higher score (53.8) than did those who were ‘Working’, either full-time or part-time, as well as those in the ‘Other’ category (unemployed/student/disability/homemaker). Two other variables were also found to have significant differences: neighbourhood longevity and dwelling type. Long-term residents living in their neighbourhoods for 20 years or more had significantly higher sense of place scores (65.3) than did those who lived in their neighbourhood for ‘Less than 5 years’ or ‘5–10 years’, or even ‘11–19 years’. With respect to dwelling type, those who lived in either a ‘Single detached’ (56.7) or a ‘Semi-detached/duplex/row/townhouse’ (56.9) were found to have significantly higher sense of place scores than did those who lived in ‘Low-rise apartment/condominium building’. Unlike the finding from the larger citywide sample, those living in a ‘High-rise apartment/condominium building’ were found to have a significantly greater sense of place score than did those who lived in a ‘Low-rise apartment/condominium building’.

The linear regression analysis resulting from Model 1 found that residents living in the Central (mixed SES) and Southwest Mountain (high SES) neighbourhoods have 9 sense of place scores higher than those who live in the Lower City (low SES), which is the reference group (Table 4). Unlike self-assessed health, mental health has a strong positive effect on sense of place. People with ‘excellent/very good’ mental health have 11 scores higher than those with ‘poor/fair’ mental health (the reference group). We also found immigrant status has a negative effect on sense of place. Those who are not born in Canada (immigrants) have a −2 score lower than those who were born in Canada. Also, the longer one resides in their neighbourhood, the higher the sense of place. Those who lived in the neighbourhood more than 10 years have approximately 9 scores higher than those who lived in the neighbourhood less than 5 years. We can also see that sense of place is strongly related to the type of dwelling. Residents living in a single detached home or semi-detached/duplex/townhouse have approximately 9 scores higher than those living in the high-rise apartment/condo buildings (the reference group).

**Table 4:** Ordinary least squares regression of sense of place in Hamilton

	Citywide sample (*n* = 1,002)		Lower city (*n* = 293)	
Adj. *R* ^2^	21.94 %		12.54 %	
Variable	Coeff.	*Z* value	Coeff.	*Z* value
*Neighbourhood cluster*				
1. Southwest Mountain	8.617***	6.16		
2. Central	9.002***	5.9		
3. Lower City (ref.)				
*Gender*				
1. Male (ref.)				
2. Female	1.527	1.47	−1.132	−0.49
*Age*				
1. 18–29 (ref.)				
2. 30–44	−0.006	0	−0.068	−0.02
3. 45–64	0.820	0.43	0.612	0.16
4. 65 or over	2.692	0.93	5.807	1.05
*Self-perceived health*				
1. Excellent/very good	1.103	0.63	1.141	0.31
2. Good	1.023	0.58	3.798	1.18
3. Fair/poor (ref.)				
*Self-perceived mental health*				
1. Excellent/very good	11.393***	5.47	10.915***	2.87
2. Good	6.571***	3.1	5.779	1.51
3. Fair/poor (ref.)				
*Born in Canada*				
1. Yes (ref.)				
2. No	−1.904*	−1.76	−2.047	−0.79
*Marital status*				
1. Married/common law	−0.146	−0.1	0.924	0.32
2. Widowed/sep./divorced	2.137	1.18	1.657	0.5
3. Single, never married (ref.)				
*Education level*				
1. Less than high school (ref.)				
2. Completed high school	0.856	0.41	3.092	0.86
3. Some college or university	−0.540	−0.24	−0.683	−0.17
4. Completed college	0.307	0.14	1.745	0.43
5. Completed bachelor	−1.416	−0.67	0.855	0.23
6. Post-graduate/professional	−2.040	−0.85	−7.553	−1.39
*Employment status*				
1. Working full-time	0.356	0.24	−4.070	−1.39
2. Working part-time	−2.383	−1.18	−6.478*	−1.75
3. Retired	5.858	2.54	0.543	0.13
4. Other (unemployed/student disability/homemaker) (ref.)				
*Lived in neighbourhood*				
1. Less than 5 years (ref.)				
2. 5–10 years	4.511***	3.02	0.855	0.26
3. 11–19 years	9.270***	6.86	6.784**	2.06
4. 20 years or more	8.376***	5.08	10.900***	3.24
*Housing tenure*				
1. Own	−0.956	−0.58	−2.118	−0.56
2. Rent (ref.)				
*Dwelling type*				
1. Single detached	9.398***	4.24	3.406	0.73
2. Semi-detached/duplex/row/townhouse	9.929***	4.39	5.090	1.07
3. Low-rise apt/condo bldg	4.479*	1.82	−5.525	−1.06
4. High-rise apt/condo bldg (ref.)				

The joint test of education of the sample of Lower City is not significant

*** Significant level at 1 %, ** at 5 % and * at 10 %

Bold items are statistically significant

When the sample is restricted to the Lower City (Model 2), the significance of the effect of dwelling type is lost. This may be because high-rise apartment/condo buildings are more concentrated in the Lower City. Similar to Model 1, the length of residence in the neighbourhood has a strong positive effect on sense of place. Although residents of the Lower City have a comparatively lower sense of place compared to the Central and Southwest Mountain neighbourhoods, those who have lived there a long time (e.g. more than 20 years) have a much higher sense of place score (10.9) than the reference group—residents who have lived in the neighborhood less than 5 years. This may be the result of the highly transient nature of the population in the Lower City. In both the entire sample (Model 1) as well as that specific to the Lower City (Model 2), socio-demographic factors appear to have no significance; age, sex, education level, employment status and marital status do not have an influence on sense of place.

The logistic regression analysis tells us that the residents living in the Southwest Mountain and Central neighbourhoods (OR = 2.3 and 2.8 respectively) are more likely to have high ‘health-related sense of place’ than those living in the Lower City (Table 5). Residents with higher education and living longer in the neighbourhood are more likely to have ‘excellent/very good’ health-related sense of place. Compared with the results in Table 4, the significance of the variable ‘education level’ indicates that residents with higher education tend to have ‘excellent/very good’ health-related sense of place. Dwelling type is also a very important factor. Consistent with the findings in Table 4, residents living in single detached housing are approximately 4 times more likely to have high ‘health-related sense of place’ than those living in high-rise apartment/condo buildings, and 2.6 times more likely than those residing in semi-detached/duplex/townhouses.

**Table 5:** Logistic regression of high health-related sense of place in Hamilton

	Citywide sample (*n* = 1,002)		Lower city (*n* = 293)	
Pseudo *R* ^2^	10.59 %		14.57 %	
Variable	Odds ratio	*Z* value	Odds ratio	*Z* value
*Southwest cluster*				
1. Mountain	2.298***	4.19		
2. Central	2.848***	4.98		
3. Lower City (ref.)				
*Gender*				
1. Male (ref.)				
2. Female	0.929	−0.44	0.869	−0.35
*Age*				
1. 18–29 (ref.)				
2. 30–44	1.088	0.27	1.322	0.38
3. 45–64	0.989	−0.04	0.731	−0.43
4. 65 or over	1.026	0.06	1.002	0
*Born in Canada*				
1. Yes (ref.)				
2. No	0.836	−1	0.715	−0.58
*Marital status*				
1. Married/common law	1.127	0.49	1.028	0.06
2. Widowed/sep./divorced	0.840	−0.63	0.896	−0.18
3. Single, never married (ref.)				
*Education level*				
1. Less than high school (ref.)				
2. Completed high school	2.436**	2.58	8.399***	2.64
3. Some college or university	2.096*	1.98	2.563	0.96
4. Completed college	2.695***	2.87	3.634	1.55
5. Completed bachelor	2.926***	3	5.294**	1.96
6. Post-graduate/professional	3.627***	3.41	4.969	1.59
*Employment status*				
1. Working full-time	1.411	1.42	1.580	0.79
2. Working part-time	1.234	0.67	1.077	0.09
3. Retired	1.725	1.39	2.567	1.13
4. Other (unemployed/student disability/homemaker) (ref.)				
*Lived in neighbourhood*				
1. Less than 5 years (ref.)				
2. 5–10 years	1.467	1.62	0.794	−0.44
3. 11–19 years	1.883***	2.72	0.568	−0.87
4. 20 years or more	1.734**	2.07	1.341	0.5
*Housing tenure*				
1. Own	1.146	0.59	0.970	−0.05
2. Rent (ref.)				
*Dwelling type*				
1. Single detached	3.895***	3.37	10.771***	2.73
2. Semi-detached/duplex/row/townhouse	2.621**	2.28	4.647	1.48
3. Low-rise apt/condo bldg	2.021	1.51	1.468	0.43
4. High-rise apt/condo bldg(ref.)				

*** Significant level at 1 %, ** at 5 % and * at 10 %

Bold items are statistically significant

The analysis of the sample comprising the Lower City yielded similar results. Residents living in single detached housing are approximately 11 times more likely to have high ‘health-related sense of place’ than those living in high-rise apartment/condo buildings. This underscores the connection between housing and health. Housing appears to be the most important variable in determining health-related sense of place, even possibly showing greater importance than the neighbourhood itself.

## Discussion

As with our earlier work (Williams et al. 2010), sense of place was found to be higher in Southwest Mountain, as well as among seniors, the retired, and long-term residents. Southwest Mountain is an upper middle class suburban neighbourhood whereas the Central and Lower City are comparatively older, lower-income neighbourhoods. Generally, those living in higher SES neighbourhoods have a higher sense of place than do those in lower SES communities. SES is also reflected in the significant differences that exist in housing tenure with those owning their homes having a higher sense of place than those who rent. Similarly, those who are fortunate to be able to afford a single-detached dwelling also have a higher sense of place than those who live in less spacious (or perhaps in some cases less private) dwelling types. Although surpassed by retired people (discussed below), those employed full time and thereby making a good wage were found to have a higher sense of place score than those in the ‘Other’ category (defined as unemployed/student/disability/homemaker).

Second, among the socio-demographic variables for the entire city sample, age (65 years and over) and length of residency (20 years or more) were found to be significantly related to sense of place. Consistent with our earlier research (Williams et al. 2010), these findings specific to age and length of residency suggest that sense of place has a temporal dimension; as people age and/or spend more time in the same neighbourhood, they are likely to have a strong sense of place. The importance of age is reflected in the significant differences that exist in employment status, with those retired (often 65 years of age and over) having the highest sense of place scores when compared to the three other employment categories. These results are in keeping with the findings of researchers who have determined that age and longevity of residence have a positive effect on sense of place (e.g. Hay 1998). This corresponds with Tuan’s (1974, p. 99) assertion that “…a person in the process of time invests bits of his emotional life in his home and beyond the home in his neighborhood”.

With respect to the findings in the three neighbourhood clusters, it is likely that the concept of self-selection bias plays an important role in perceptions of sense of place. It can be assumed that the longer a person lives in their neighbourhood and as they age, they will grow accustomed to their surroundings and their neighbours. This is particularly the case in higher income neighbourhoods, such as Southwest Mountain, where residents have the means to move if they are unhappy. As a result, long-term residents are more likely to report being satisfied with where they live. This is captured in Table 2 where the second largest gap in sense of place scores (after mental health) is years lived in the neighbourhood. By comparison, residential choice may be severely limited in lower-income communities such as the Lower City. In fact, as noted in our earlier work, this neighbourhood has an average housing value about half that found in Southwest Mountain, a substantially lower median household income, and an unemployment rate twice as high. Lower housing values, coupled with job losses in the manufacturing and service sectors in Hamilton, constrain residents of lower income neighbourhoods, such as Lower City, in their ability to move if they are unhappy with their current location. These socio-economic issues have likely contributed to a lower sense of place among some residents.

There are a number of possible explanations as to why unmarried people (widowed/separated/divorced) are found to have a higher sense of place than did those who were ‘single, never married’. This finding is in keeping with the literature addressing sense of community, where families with young children (even if separated, divorced and widowed) are found to have a stronger sense of community than are those who are not married and without children (e.g. Nasar and Julian 1995; Robinson and Wilkinson 1995). It is possible that issues related to work-life demands and daily activity patterns in an automobile dominated society have influenced neighbourhood perceptions among single, never-married people. This group likely spends considerable time away from their neighbourhood, engaging in a variety of daily activities and as a result, has less of a connection to it. As suggested by Relph (2007), for some people, sense of place is weak because they are focused on non-place based interests such as leisure activities and career advancement: “places for [these individuals] are little more than incidental backgrounds to other concerns…” (Relph 2007, p. 19).

The analysis found that unlike education, employment status had an impact on sense of place. Although these two variables often mirror one another, only employment status was significant. Specifically, retired people had a much higher sense of place compared to those who were employed. We can speculate that sense of place is strongly influenced by the degree of experiences and opportunities offered to residents. It is likely that people who are employed have a somewhat wider *geographic scope* with respect to what constitutes their community and tend to regard *place* more broadly, whether, for example, related to their city or region (rather than the immediate area in which they reside). By comparison, as a result of a different set of experiences and opportunities related to socio-economic status and social networks, retired people and those in the unpaid workforce (in the case of the Lower City, see Table 3) may have a geographic frame of reference that is more likely to be oriented to the neighbourhood in which they live. As a result of these unique factors, a certain ‘comfort zone’ sets in where residents enjoy a more stable lifestyle that manifests itself in a higher sense of place.

Unlike our earlier work in Hamilton (Williams et al. 2010), the current analysis found that gender and immigrant status did not influence sense of place. Consistent with our earlier research, the citywide analysis found a significant difference between owners and renters, with owners having a significantly higher sense of place than the renters. This makes intuitive sense given that homeowners are interested in investing in where they live, not only because of pride but also as a matter of practicality as they want to be sure that their home either sustains or increases its value over time. As suggested by Frumkin (2003) and Billig (2005), differences in population characteristics and urban form can also influence perceptions of sense of place. Southwest Mountain’s location and size means that residents have access to open spaces, parks, and to a variety of community and recreational services—all which contribute to an enhanced sense of place. Conversely, the Central and Lower City neighbourhoods have comparatively higher densities, are made up of older homes, have a more transient population, and have less park space and community services; these characteristics are likely to diminish sense of place.

Consistent with our earlier work (Williams et al 2010), the results of this paper point to the significance of age and length of residency in influencing sense of place. Determining the importance of sense of place for the quality of life of seniors who are ‘aging-in-place’ appears to be a practical application for moving the research forward. Examining the sense of place of this particular sub-group may shed light on the experience of aging-in-place, which is in keeping with society’s current focus on community-based care for this cohort. Other neighbourhood-specific interventions which could potentially improve the community’s sense of place, which may ultimately lead to improved health outcomes, if not overall well-being of all residents. Sense of place interventions can be wide-ranging, including the establishment of neighbourhood associations, the organization of block parties and yard sales. Advocating for improved recreational facilities and services or investing into the welfare of the community as a whole could also work to increase residents’ sense of place.

Finally, the paper examined health-related sense of place. That is, people who have a strong sense of place and who also rate their health and mental health positively. The results of this analysis (Table 5) showed that education and housing were strongly associated with this outcome. In particular, people living in single detached homes were far more likely to have high health-related sense of place. While the Lower City is a relatively disadvantaged area with older housing, the analysis revealed that residents of this neighbourhood who lived in single detached homes enjoyed an especially strong health-related sense of place. This finding underscores the importance of housing as a social determinant of health and by extension sense of place. While an extensive literature in geography and other disciplines has explored the issues associated with health and housing (e.g. Dunn 2000; Bryant 2003; Shaw 2004) more attention could be focused on how the type of dwelling (in terms of its physical characteristics) may influence perceptions of health and place.

## Next Steps

Further advances in understanding the effects of contextual (or place-based) and compositional (or individual-level) factors on health are likely to be made by studies using primary data to investigate specific processes through which perceptions of place impact health outcomes. Such studies would need to incorporate direct measures of individuals’ perceptions of place along with individual and place-based characteristics and examine interactions such as the relation of the place-based socio-economic context to potential mediators of place effects. Related to this, future research can be directed at assessing how neighbourhood form and design influences residents’ perceptions; for example, factors such as density, housing type, street layout and placement of parks and recreational services can be examined. Furthermore, the issue of sense of place among residents of ‘sprawl communities’ is particularly pressing given the growth of suburbs in North American. The limited recent research in this area (Frumkin et al 2004; Sturm and Cohen 2004; OCFP 2005) suggests that urban sprawl may have a negative impact on residents’ sense of place given the greater feelings of isolation and related mental and physical health concerns.

## Footnotes

1 For example, if a survey respondent gave positive answers (Code 1) on each of the four questions then his/her total is 4. Subtracting this number from 20 (20 − 4) gives the respondent a new score of 16, representing the best possible outcome. Conversely, if a respondent gave negative answers (Code 5) on each of the four questions, then his/her total is 20. Subtracting 20 from this number (20 − 20) gives the respondent a new score of 0, the lowest possible outcome. Thus a new scale is created on each dimension of sense of place ranging from 0 to 16.
